# Inhibition of DNA methyltransferase activity and expression by treatment with the pan-deacetylase inhibitor panobinostat in hepatocellular carcinoma cell lines

**DOI:** 10.1186/1471-2407-12-386

**Published:** 2012-09-03

**Authors:** Steffen Zopf, Matthias Ocker, Daniel Neureiter, Beate Alinger, Susanne Gahr, Markus F Neurath, Pietro Di Fazio

**Affiliations:** 1 Department of Medicine 1, University Hospital Erlangen, Ulmenweg 18, Erlangen, 91054, Germany; 2 Institute of Pathology, Paracelsus Medical University, Salzburg, Austria; 3 Department of Pneumology, Klinikum Nuremberg Nord, Nuremberg, Germany; 4 Institute for Surgical Research, Philipps University Marburg, Marburg, Germany

**Keywords:** DNA methyltransferase, Deacetylase inhibitor, Epigenetics, Transcriptional control, Hepatocellular carcinoma

## Abstract

**Background:**

Hepatocellular carcinoma (HCC) still represents an unmet medical need. Epigenetic inactivation of tumor suppressor genes like RASSF1A or APC by overexpression of DNA methyltransferases (DNMTs) has been shown to be common in HCC and to be linked to the overall prognosis of patients. Inhibitors of protein and histone deacetylases (DACi) have been demonstrated to possess strong anti-tumor effects in HCC models.

**Methods:**

We therefore investigated whether DACi also has any influence on the expression and activity of DNMTs and methylated target genes in HepG2 and Hep3B cell culture systems and in a xenograft model by immunohistochemistry, westernblotting, RT-qPCR and methylation-specific PCR.

**Results:**

Our findings demonstrate a rapid inhibition of DNMT activity 6 h after treatment with 0.1 μM of the pan-DACi panobinostat. A downregulation of DNMT mRNAs and protein were also observed at later points in time. This loss of DNMT activity and expression was paralleled by a diminished methylation of the target genes RASSF1A and APC and a concomitant re-expression of APC mRNA and protein. Analysis of HepG2 xenograft specimens confirmed these results in vivo.

**Conclusion:**

We suggest a dual mode of action of DACi on DNA methylation status: a rapid inhibition of enzyme activity due to interference with posttranslational acetylation and a delayed effect on transcriptional control of DNMT genes by HDAC or miRNA mechanisms.

## Background

Hepatocellular carcinoma (HCC) is the most common primary tumor of the liver and represents an unmet medical need, being among the most common tumor diseases and causes of cancer related deaths worldwide and showing a rising incidence also in Western countries [1-3]. Although the multi-kinase inhibitor sorafenib has recently been approved for treatment of advanced stage HCC, the overall efficacy still remains dissatisfying [4].

Besides genetic alterations, changes in chromatin have recently been identified to contribute to tumorigenesis [5]. These reversible modifications are considered to contribute to tumor suppressor gene inactivation by means of DNA methylation, histone modifications or miRNA expression. Expression of DNA methyltransferases (DNMT) has been shown to be associated with liver cancer formation and DNA hypermethylation [6,7], especially in the presence of hepatitis B or hepatitis C viruses [8-10] and has been linked to poor prognosis [11]. Today, three DNMTs have been identified in human cells. While DNMT1 methylates newly synthesized DNA during cell division (maintenance DNMT), DNMT3a and DNMT3b act on methylation of CpG motifs during cellular differentiation and regulatory processes (de novo DNMT) [12]. Genes that are commonly affected by DNA methylation include both the tumor suppressors RASSF1A (Ras association domain family 1 isoform A) and also APC (adenomatous polyposis coli). Both genes have been shown to be commonly inactivated in human hepatocellular carcinoma and to influence the overall prognosis of patients and therefore represent interesting targets for reversing DNA methylation status [13-17].

Besides DNA methylation, post-translational modifications such as acetylation, SUMOylation or phosphorylation occurring at amino acid residues in histone proteins have also been identified as strong epigenetic regulators of gene transcription. Previously, we have shown that expression of histone deacetylases (HDAC) is significantly associated with HCC grading and that HDAC2 represents an independent prognostic factor in HCC [18]. While inhibition of HDAC is usually attributed to transcriptional control of cell cycle regulators like p21^cip1/waf1^[19], additional effects involving non-nuclear protein modifications have recently been described, e.g. the interaction with chaperones such as heat shock protein 90 (HSP90) [20,21]. Although these cellular targets of deacetylases are not well known today, some reports confirm a transcriptional control of DNMT by HDAC [22,23].

Panobinostat is a novel orally available pan-deacetylase inhibitor with broad anti-tumor activity [24]. Our own previous results showed a significant inhibition of HCC growth in vitro and in xenograft models in vivo which were mediated by alternative pathways of apoptosis induction such as activation of the unfolded protein response [25]. We therefore investigated whether panobinostat also influences the activity of DNMT in HCC cell lines and if this affects the expression and methylation status of CpG promoter islands of known tumor suppressor genes in HCC models. We can show here that panobinostat exerts a dual effect on DNMT activity and expression, indicating that deacetylase inhibitors can also indirectly control DNA methylation status.

## Methods

### Cell culture

The human hepatocellular carcinoma cell lines HepG2 (p53^wt^) and Hep3B (p53^−/−^) were cultured on six-well tissue culture plates (Becton Dickinson, Mannheim, Germany) in RPMI-1640 (Biochrom, Berlin, Germany) or Dulbecco’s modified Eagle’s medium (DMEM, Biochrom, Berlin, Germany) containing 10% fetal calf serum (Biochrom), penicillin (107 U/l) and streptomycin (10 mg/l) at 37 °C in an atmosphere containing 5% CO2. All cell lines were obtained from the German Collection of Microorganisms and Cell Cultures (DSMZ, Braunschweig, Germany). Cells were starved for 24 h in medium containing 0.125% FCS to achieve cell cycle synchronization and then washed twice with phosphate-buffered saline (PBS; Biochrom), treated with trypsin EDTA (0.05% Trypsin, 0.02% EDTA; Biochrom), seeded at a density of 0.5x10^6^ per well [25]. Panobinostat was a gift from Novartis Pharma AG, Basel, Switzerland, and was dissolved in dimethylsulfoxide (DMSO; Sigma, Deisenhofen, Germany) and then further diluted with culture medium [25]. Cells were treated with 0.1 μM panobinostat for 6 to 72 h and then processed for further analyses.

### HepG2 xenograft samples

Samples from previously established xenografts of HepG2 cells to male athymic nu/nu NMRI mice (Harlan Winkelmann GmbH, Borchen, Germany) were used for this study [25]. HepG2 cell lines were harvested and resuspended in sterile physiologic NaCl solution. 5.0 × 10^6^ cells were injected subcutaneously into the flank of 6 to 8 week old male mice (mean weight 38.2 +/− 4.3 g). Eight animals were used for each treatment group. Animals were kept in a light and temperature controlled environment and provided with food and water *ad libitum*. Tumor size was determined daily by measurement using a caliper square. When subcutaneous tumors reached a diameter of 7 mm, daily i.p. treatment with panobinostat (10 mg/kg) or vehicle (physiologic saline solution) was started. Animals were sacrificed by cervical dislocation and tumor samples collected after 1, 7 and 28 days of treatment or when reaching the termination criteria (e.g. weight loss > 20%, tumor diameter > 25 mm or tumor ulceration through the skin). Tumor and tissue samples were fixed in 10% phosphate-buffered formalin or snap-frozen in liquid nitrogen. All animals received humane care. The study protocol complied with the institute’s guidelines and was approved by the Government of Lower Franconia (Würzburg, Germany, file number 54–2531.31-3/06) prior to the commencement of the experiments. Hep3B cells proved not to be tumorigenic in NMRI mice and were therefore not used for in vivo experiments.

### Measurement of DNMT activity

Nuclear protein was isolated with EpiQuik™ Nuclear Extraction Kit I (Epigentek, Brooklyn, NY, USA) from cells exposed to panobinostat or from untreated control cells. After protein quantification with Total Protein Kit (Micro Lowry, Peterson’s Modification; Sigma-Aldrich Chemie GmbH, Munich, Germany), 12 μg of nuclear protein was used to measure total DNMT activity with the EpiQuik™ DNA Methyltransferase Activity/Inhibition Assay (Epigentek) in accordance with the manufacturer’s instructions.

### Isolation of total RNA and quantitative real-time RT-PCR

Total cellular RNA was extracted using the RNeasy Kit (Qiagen, Hilden, Germany) in accordance with the manufacturer’s instructions. Reverse transcription into cDNA was performed using Superscript III RNAse H-reverse transcriptase (Live Technologies, Darmstadt, Germany) with dT15 (TIB-Biomol, Berlin, Germany) and random hexamer primers (Promega, Heidelberg, Germany) as previously described [26]. QuantiTect Primers for DNMT1, DNMT3a, DNMT3b, APC, RASSF1A and GAPDH were purchased from Qiagen and subjected to quantitative real-time RT-PCR on a LightCycler system (Roche Molecular Biochemicals, Mannheim, Germany) using the LightCycler FastStart DNA Master SYBR Green I Kit (Roche Molecular Biochemicals). Results were analyzed with the LightCycler software and normalized to GAPDH mRNA content for each sample.

### Quantitative methylation-specific real-time PCR

Total DNA was extracted from cell culture samples and tissue specimens from nude mice by using the DNeasy Blood and Tissue Kit (Qiagen). DNA was then subjected to sodium bisulfate conversion using the EpiTect Bisulfite Kit (Qiagen). Bisulfite converted DNA was then used to perform a quantitative methylation-specific PCR (qMSP) with primers and TaqMan probes (see Table 1) specific for nucleotide sequences containing methylated cytosines at CpG positions [27]. qMSP was performed using the EpiTect MethyLight PCR Kit (Qiagen) in accordance with the manufacturer’s instructions.

**Table 1:** Primer and TaqMan probe sequences for qMSP


**APC**	
Forw.	5Â´-AGTGCGGGTCGGGAAGC-3Â´
Rev.	5Â´-AACCACATATCGATCACGTACG-3Â´
TaqMan-Probe:	5Â´-FAM-AAAACGCCCTAATCCGCATCCAACG-TAMRA-3Â´
PCR parameter:	Denaturation: 95°C, 10 min. (1 cycle), Amplification: 95°C, 10 sec.; 64°C, 60 sec. (50 cycle); Denaturation: 40°C, 30 sec. (1 cycle)
**RASSF1A**	
Forw.	5Â´-GCG TTG AAG TCG GGG TTC-3Â´
Rev.	5Â´-CCC GTA CTT CGC TAA CTT TAA ACG-3Â´
TaqMan-Probe:	5Â´-FAM-ACAAACGCGAACCGAACGAAACCA-TAMRA-3M
PCR parameter:	Denaturation: 95°C, 10 min. (1 cycle), Amplification: 95°C, 10 sec.; 63°C, 60 sec. (50 cycle); Denaturation: 40°C, 30 sec. (1 cycle)

### Protein extraction and Westernblot analysis

Whole cell lysates were prepared from panobinostat-treated cells, untreated controls and xenograft tissue samples as previously described [28]. Total protein was extracted from cultured cells by adding 2X sample buffer (10 mM sodium chloride, 0.5% Nonidet NP40 (Amresco, Solon, OH, USA), 20 mM Tris–HCl pH 7.4, 5 mM magnesium chloride, 10 μg/ml complete protease inhibitor cocktail (Roche Diagnostics, Mannheim, Germany), 1 mM phenylmethylsulfonylfluoride). DNA was shared by pipetting up and down for 3 minutes at room temperature. Samples were boiled at 95°C for 15 minutes, centrifuged at 13,000 rpm for 10 seconds and then subjected to 14% SDS-PAGE (Life Technologies). After blocking overnight at 4°C in a buffer containing PBS, 0.1% Tween-20 and 5% low fat milk powder, nitrocellulose membranes were incubated for 90 minutes with primary antibodies. Antibodies against DNMT1, DNMT3a, DNMT3b (Cell Signaling Technology, Denvers, MA, USA), APC, RASSF1A (both from Abcam, Cambridge, UK) and β-actin (Sigma, Deisenhofen, Germany) were used. Membranes were washed three times for 10 minutes in a buffer containing PBS and 0.1% Tween-20 and were incubated with a peroxidase coupled secondary antibody (1:1,000, Pierce, Rockland, IL, USA) to visualize responsive bands after incubation with West Pico luminescence substrate (Pierce). Densitometry analysis was performed by peak intensity analysis on a GeneGnome image capture and analysis system (GeneGnome, Syngene, UK). Bands were normalized to β-actin expression which was used as an internal loading control.

### Immunohistochemistry

Formalin-fixed and paraffin-embedded xenograft tumour samples were cut into 5 μm sections deparaffinised using graded alcohols. Antigen retrieval was performed by heat-induced epitope retrieval in pH = 9 antigen retrieval buffer (Dako, Glostrup, Denmark) at 95°C for 60 minutes. Endogenous peroxidase blocking was carried out for 10 minutes with peroxidase blocking reagent (Dako). Subsequently, the primary antibody against DNMT1 (monoclonal, mouse anti-human, Antibodies-Online, Aachen, Germany, ABIN466002, 1:50) and DNMT3a (polyclonal, rabbit anti-human, Santa Cruz Biotechnology, Heidelberg, Germany, sc-20703, 1:200) was applied for 30 minutes at RT. For detection of the primary antibodies the ready-to-use REAL™ EnVision™ Detection System (Dako) was used in accordance with the manufacturer’s instructions. The EnVision staining system is based on an HRP labeled dextran polymer, which is conjugated to secondary antibodies eliminating the nonspecific staining background resulting from endogenous avidin-biotin activity. Visualization was performed using diaminobenzidine (DAB) as the chromogen substrate being a part of the REAL™ EnVision™ Detection System. Slides were counterstained with hematoxylin. The stained slides were digitalized using the ImageAccess 9 Enterprise software (Imagic Bildverarbeitung, Glattbrugg, Switzerland). The percentage numbers of DNMT1 and DNMT3a nuclear expressing tumor cells were evaluated for the 3 different high power fields (400 magnification) using the particle analysis module with the optimized binarisation method of the image analysis system.

### Statistical analysis

Statistical analysis was performed using SPSS 15.0.1 for Windows (SPSS Inc., Chicago, IL, USA). Significance was calculated using the t-test for paired samples. P < 0.05 was regarded as significant (*).

## Results

### Panobinostat inhibits DNMT activity and expression in vitro

**Figure 1:**
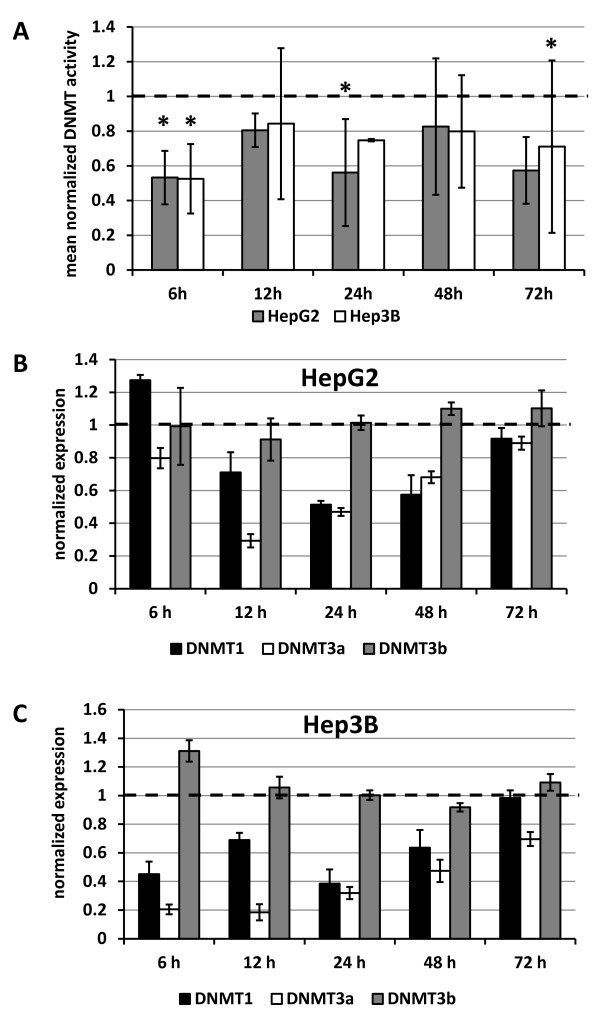
**Panobinostat affects activity and expression of DNA methyltransferases *in vitro*.** (**A**) Total DNMT activity was evaluated in HepG2 and Hep3B cells treated with 0.1 μM panobinostat for the indicated points in time. Results are the mean remaining DNMT activity ± relative error of three independent experiments and are expressed relative to values of untreated controls with a set value of 1.0 for each point in time. * *P* < 0.05 vs. untreated controls. (**B**) and (**C**) Quantitative RT-PCR analysis of expression of DNMTs in HepG2 (**B**) and Hep3B (**C**) cells after treatment with 0.1 μM panobinostat. Results were normalized to the GAPDH level of each sample and represent mean ± relative error of three independent experiments and are expressed relative to mRNA levels of untreated controls at each point in time using the set value 1.0.

After only 6 h of treatment, incubation of HepG2 and Hep3B cells led to a rapid and significant decrease in total DNMT activity by 46.7% and 47.4%, respectively. At later points in time, DNMT activity was stably reduced by approximately 20% in both cell lines, except for the 24 and 72 h time point in HepG2, where an inhibition of more than 40% was observed (Figure 1A).

**Figure 2:**
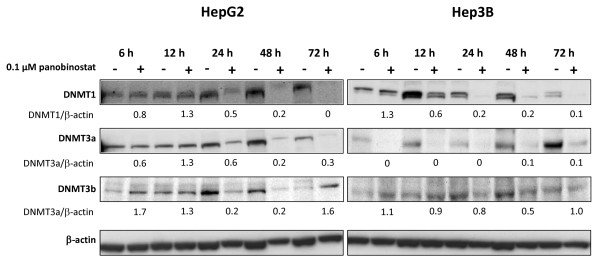
**Westernblot analysis of DNMT expression.** HepG2 and Hep3B cells were incubated with 0.1 μM panobinostat for the indicated points in time. Western blot results show representative examples for expression of DNMT1, DNMT3a and DNMT3b as well as β-actin which served as an internal control. Densitometry values are relative to untreated controls with a set value of 1.0 for each point in time.

Expression of DNMT1, DNMT3a and DNMT3b were then investigated by quantitative real-time RT-PCR. Panobinostat treatment significantly repressed mRNA for DNMT1 and DNMT3a in both cell lines while no changes were observed in DNMT3b levels (Figure 1B/C). These findings were corroborated by westernblot analysis showing a strong reduction of DNMT1 and DNMT3a protein in both cell lines but not of DNMT3b (Figure 2). Here, only a transient decrease in protein levels was observed after 24 to 48 h in both cell lines. Although mRNA levels in total were rapidly decreased by panobinostat, protein expression was significantly reduced after only 24 h and remained suppressed until 72 h for DNMT1 and DNMT3a.

### Effects of panobinostat on target gene methylation and expression in vitro

**Figure 3:**
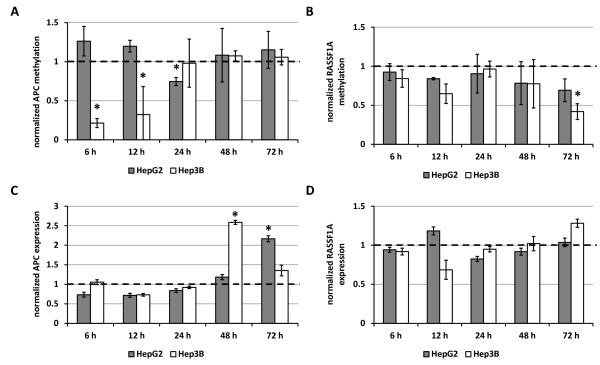
**Regulation of DNA methylation and expression of target genes after panobinostat treatment.** DNA methylation of APC (**A**) and RASSF1A (**B**) was detected by quantitative methylation-specific PCR in HepG2 and Hep3B cells treated with 0.1 μM panobinostat. Expression of total mRNA for APC (**C**) and RASSF1A (**D**) was analyzed using quantitative real-time RT-PCR and normalization to GAPDH content of each sample. Results are mean ± relative error of three independent experiments and are expressed relative to the untreated controls with a set value of 1.0. * *P* < 0.05 vs. untreated controls.

We next investigated whether the inhibition of DNMT activity and expression is also reflected on the methylation pattern of known hypermethylated tumor suppressor genes. In order to do so, quantitative methylation specific PCR was performed for APC and RASSF1A in cells treated with 0.1 μM panobinostat for 6 to 72 h and expressed relative to the levels of untreated controls at the given points in time (Figure 3). Overall, Hep3B cells seemed to be more sensitive to the DACi-mediated inhibition of DNA methylation as shown by a significant and strong reduction of methylated APC after only 6 h. While methylation was suppressed by approximately 80% here, APC methylation returned to the level of untreated controls after 24 h. RASSF1A showed a slight reduction in methylation at 12 h but only proved to be significant (60% reduction) at 72 h. In HepG2, APC methylation was significantly reduced after only 24 h of treatment while no change was observed for RASSF1A. In line with the reduction of methylation, an increased expression of APC was observed in both cell lines, reaching the highest level at 48 h for Hep3B and at 72 h for HepG2, respectively (Figure 3C). Observation of methylation of RASSF1A showed no significant change in expression induced by panobinostat (Figure 3D).

### Panobinostat influences methylation and gene expression pattern in vivo

**Figure 4:**
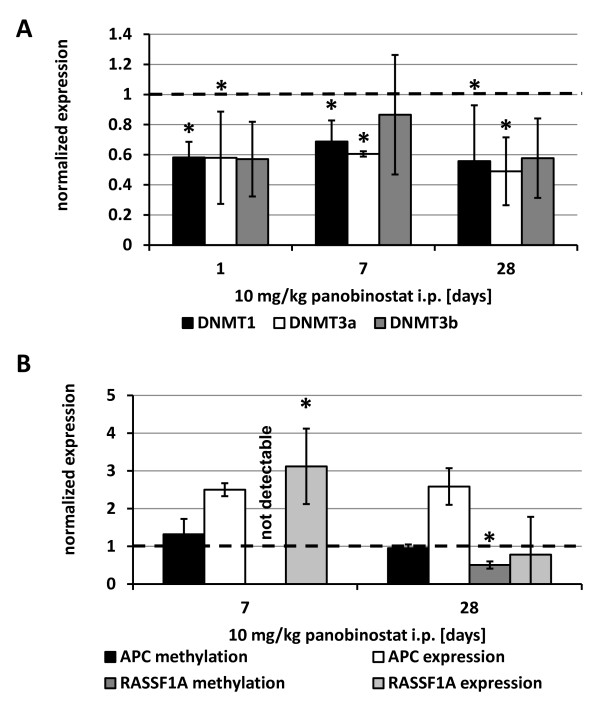
**Effect of panobinostat on DNMT and target gene expression *in vivo*.** HepG2 xenograft specimens were analyzed for expression of (**A**) DNMTs after 1, 7 and 28 days of daily i.p. injections of 10 mg/kg panobinostat. Results were normalized to the GAPDH content of each sample and represent mean ± relative error of 5 to 10 independent samples per group and are expressed relative to expression levels of untreated control animals with the set value of 1.0 for each point in time. (**B**) Methylation status and total expression level of APC and RASSF1A were analyzed at day 7 and day 28 of panobinostat treatment. Results are normalized to levels of untreated controls. Methylation of RASSF1A was not detectable in untreated controls and in treated animals at day 7. * *P* < 0.05 vs. untreated controls.

To address whether panobinostat also influences expression of DNMTs and related target genes *in vivo*, we analyzed HepG2 xenograft samples from a previously described nude mouse model [25]. Animals were treated with daily intraperitoneal injections of 10 mg/kg panobinostat. After only 1 day expression of all DNMTs were reduced by approximately 40% compared to untreated controls. The observed reduction in expression was statistically significant (*P* < 0.05) for DNMT1 and DNMT3a (Figure 4A). Although expression of DNMT3b was also reduced in the *in vivo* setting, the results were not of statistical significance (*P* = 0.06, 0.28 and 0.08 at day 1, 7 and 28, respectively), and therefore confirmed the above described *in vitro* findings.

The methylation status and total mRNA expression of APC and RASSF1A were analyzed from these samples after 7 and 28 days of treatment (Figure 4B). Interestingly, while the methylation status of APC did not differ as compared to untreated controls, the expression of APC was induced 2.5-fold. Methylated RASSF1A was not detectable at day 7 in either the untreated controls or the treated animals, however, a reduction of approximately 50% was measured at the end of the study period in the treated animals as compared to the controls. Expression of RASSF1A was not elevated at this point in time but showed a significant increase at day 7.

**Figure 5:**
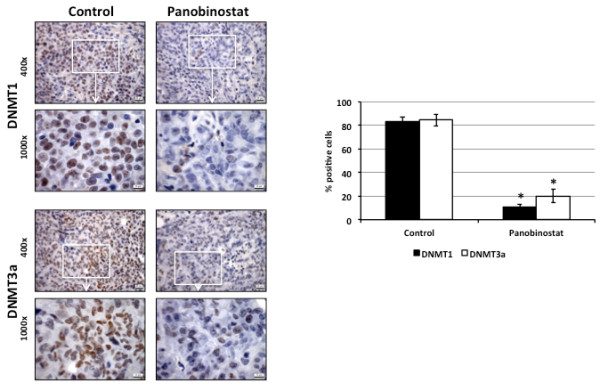
**Immunohistochemical analysis of DNMT1 and DNMT3a expression.** HepG2 xenografts were treated with daily i.p. injections of 10 mg/kg panobinostat for 28 days. Untreated controls show a high nuclear expression of both DNMTs, while a significant reduction in expression is observed in treated samples. Bar diagram shows mean percentage of positive cells ± standard deviation of n = 5 in each group. * *P* < 0.05 vs. untreated controls. Bar represents 20 μm (400× magnification) for overview and 10 μm (1000 x magnification) for detailed areas (marked by a rectangle).

These results were confirmed by immunohistochemical analyses after 28 days of treatment with 10 mg/kg panobinostat (Figure 5). Nuclear expression of both DNMT1 and DNMT3a was significantly reduced in HepG2 xenograft samples. While DNMT1 and DNMT3a were expressed in 83.3% and 84.6% of all cells in untreated controls, only 10.7% and 20.0% stained positive for these markers at the end of the treatment period.

## Discussion

Gene silencing by epigenetic mechanisms like DNA methylation or histone acetylation has been shown to contribute to HCC development [29-31]. These epigenetic mechanisms alone or in combination with genetic modifications like mutations can lead to the inactivation of tumor suppressor genes such as RASSF1A or APC and thus promote hepatocarcinogenesis [32-36]. While RASSF1A has been demonstrated to be hypermethylated in several series of clinical HCC specimens, other potential candidates such as p16 (CDKN2a), retinoic acid receptor (RAR) or H-cadherin (CDH13) are reported to be low or unmethylated and were therefore not considered to be suitable target genes for our study [30,37,38]. The reversal of epigenetically silenced genes has therefore received increasing attention recently and various studies aimed at reversing the hypermethylated or hypoacetylated phenotype in tumors. Promising preclinical results using DNMT inhibitors like 5-azacytidine, 5-aza-2’-deoxycytidine or zebularine have been obtained in HCC models [39-41]. Similarly, various histone deacetylase inhibitors, e.g. trichostatin A, SAHA, or the novel pan-deacetylase inhibitor panobinostat have been investigated in HCC cell culture and animal models showing a high efficacy in inhibiting tumor cell growth. Furthermore, we recently reported a good safety profile of panobinostat in combination with sorafenib in a patient with metastatic HCC [25,42-44]. While the classically considered mode of action of these compounds is regarded as interfering with chromatin structure and regulating the accessibility of transcriptional complexes to the DNA [19], recent evidence suggests that modifying non-histone proteins contributes to the potent effects of deacetylase inhibitors in cancer cells [25,45]. In line with this view, recent data confirms that DNMTs can also be inhibited by deacetylase inhibitors [23,46]. We have demonstrated here for the first time that treatment with the pan-deacetylase inhibitor panobinostat rapidly reduces the activity of DNMT1 and DNMT3a in two liver cancer cell lines *in vitro* after only 6 h of incubation and independent of their p53 status while the expression of these enzymes is affected only at later points in time. These data indicate that panobinostat leads to a rapid inactivation of the enzymatic function of DNMTs, probably by interfering with the protein folding and acetylation status of these proteins which is also reflected by a rapid decrease in the methylation levels of APC. This hypothesis is supported by a recent report on novel acetylation sites in lysine residues of DNMT1 that could be influenced by class III HDAC enzymes [47]. DNMT1 was also shown to be stabilized by HDAC1 mediated deacetylation and protection from proteasomal degradation, which represents a target of panobinostat, indicating a cross-dependency of acetylation and protein function [46]. Additionally, it was also demonstrated that inhibition of deacetylase function leads to ubiquitin-mediated degradation of DNMT1 and could thus also contribute to the reduced expression observed in our model [48]. The here observed delayed downregulation of DNMT mRNA and protein could also be attributed to a decreased mRNA stability as was previously demonstrated for DNMT1 and DNMT3b after treatment with Trichostatin A in Jurkat or endometrial cells [23,49]. Panobinostat was shown to downregulate DNMT1 without affecting DNMT3a and 3b in human breast cancer cells and human acute leukemia cells while we observed an additional effect on DNMT3a in the used HCC cell lines [48,50]. Here we found a downregulation of total DNMT activity and suppression of DNMT1 and DNMT3a protein expression but not of DNMT3b. In contrast to the known concept of maintenance and de novo DNMTs, it was shown that the loss DNMT1 can be compensated by DNMT3b [51,52], confirming our results of a residual DNMT activity after panobinostat treatment. These findings demonstrate divergent effects of deacetylase inhibitor treatment on individual DNMTs dependent on the cell type and the intracellular context. Additional regulatory effects responsible for this phenomenon could involve the altered miRNA profile after treatment with deacetylase inhibitors [53-55]. We have previously shown that panobinostat is a strong modulator of miRNA expression in liver cancer cell lines [56] and it was also demonstrated by others that various miRNAs, e.g. miR-29, miR-148 or miR-185, can regulate the expression of DNMTs [57-61] and thus crosslink deacetylase inhibition to mechanisms of DNA methylation [22,23,62].

Interestingly, panobinostat affects the expression of the maintenance DNMT1 and of DNMT3a, which is (together with DNMT3b) considered as a *de novo* DNA methyltransferase acting during DNA replication and cell division [12]. An overexpression of DNMTs has previously been reported in HCC, in precancerous cirrhotic lesions and in dysplasias, indicating a strong contribution of epigenetic events in HCC development [6,7,11,33,63]. In line with our previously reported data on inhibition of cell proliferation by panobinostat [25], a secondary and delayed effect on target gene methylation and reexpression was observed in both cell lines for APC at 48 and 72 h, respectively. We therefore propose a dual mode of action of pan-deacetylase inhibitors such as panobinostat on epigenetic control of gene expression: deacetylase inhibitors primarily influence the acetylation status and function of various cytosolic and nuclear proteins including DNMTs. The rapid inhibition of DNMT activity could be attributed to alterations in the protein folding due to impaired acetylation. This also influences the turnover of affected proteins and could lead to the previously described activation of the unfolded protein response and induction of non-canonical apoptosis pathways [25]. Deacetylase function also controls the acetylation status of histones which, together with DNMTs and putative miRNAs, control transcriptional processes. This not only leads to the well described upregulation of tumor suppressor genes such as p21^cip1/waf1^[19], but also to the suppression of DNMT expression and alterations in miRNA profiles [53,62] which additionally affect the translational processes leading to the desired growth-inhibitory and pro-apoptotic effects of deacetylase inhibitors in tumor cells.

## Conclusion

In summary, our data indicates that, in addition to the epigenetic activity, deacetylase inhibitors act on protein folding and function which mediates various additional effects such as activation of the unfolded protein response or transcriptional and translational control of tumor suppressor genes. Further studies are urgently required in order to better understand this multitude of effects (in particular the effects on miRNA and cellular acetylome).

## Competing interests

M.O. received honoraria and funding from Novartis Pharma AG, Nuremberg, Germany, the manufacturer of panobinostat. The other authors declare no competing interests.

## Authors’ contributions

SZ planned and performed the experiments and drafted the manuscript. PDF carried out the in vivo experiments and supervised the data. DN and BA carried out the immunohistochemistry. SG performed the RT-qPCR experiments. MFN supervised the data and drafted the manuscript. MO designed the study, supervised the data and drafted the manuscript. All authors read and approved the final manuscript.

## Pre-publication history

The pre-publication history for this paper can be accessed here:

http://www.biomedcentral.com/1471-2407/12/386/prepub
